# Gene delivery to Nile tilapia cells for transgenesis and the role of PI3K-c2α in angiogenesis

**DOI:** 10.1038/srep44317

**Published:** 2017-03-20

**Authors:** Fernanda Maria Policarpo Tonelli, Samyra Maria dos Santos Nassif Lacerda, Marcela Santos Procópio, Breno Luiz Sales Lemos, Luiz Renato de França, Rodrigo Ribeiro Resende

**Affiliations:** 1 Cell Signaling & Nanobiotechnology Laboratory, Department of Biochemistry & Immunology, Federal University of Minas Gerais, Belo Horizonte, Brazil; 2 Nanocell Institute, Divinópolis, MG, Brazil; 3 Laboratory of Cellular Biology, Department of Morphology, Federal University of Minas Gerais, Belo Horizonte, Brazil; 4 National Institute for Amazonian Research (INPA), Manaus, AM, Brazil

## Abstract

Microinjection is commonly performed to achieve fish transgenesis; however, due to difficulties associated with this technique, new strategies are being developed. Here we evaluate the potential of lentiviral particles to genetically modify Nile tilapia cells to achieve transgenesis using three different approaches: spermatogonial stem cell (SSC) genetic modification and transplantation (SC), *in vivo* transduction of gametes (GT), and fertilised egg transduction (ET). The SC protocol using larvae generates animals with sustained production of modified sperm (80% of animals with 77% maximum sperm fluorescence [MSF]), but is a time-consuming protocol (sexual maturity in Nile tilapia is achieved at 6 months of age). GT is a faster technique, but the modified gamete production is temporary (70% of animals with 52% MSF). ET is an easier way to obtain mosaic transgenic animals compared to microinjection of eggs, but non-site-directed integration in the fish genome can be a problem. In this study, PI3Kc2α gene disruption impaired development during the embryo stage and caused premature death. The manipulator should choose a technique based on the time available for transgenic obtainment and if this generation is required to be continuous or not.

Fish transgenesis has received attention since the early 1980s, when Mclean and Talwar genetically modified rainbow trout (*Oncorhynchus mykiss*) by microinjecting newly fertilised eggs^1^. Although microinjection is the conventional method for transgenesis, besides requiring highly trained personnel, it is associated with some difficulties; for example, identification of the pronuclei of the fertilised fish eggs (invisible in opaque fish eggs), microinjection through the hard chorion present in some fish species^2^, and also a low efficiency rate of genomic integration^3^,^4^,^5^. Therefore, new strategies are being developed in order to achieve fish transgenesis. Here we analyse three strategies based on lentiviral particles as gene delivery vehicles: newly fertilised egg transduction, *in vivo* transduction of spermatozoa in adult fish testis, and *in vitro* genetic modification of spermatogonial stem cells (SSCs) followed by their transplantation into Nile tilapia larvae. Researchers have used these techniques to generate transgenic mice and rats^6^,^7^,^8^,^9^. ,also the infection of in vitro cultured sperm was already reported in fish, specifically zebrafish^10^. However, in studies by Kurita *et al*., the authors used a retrovirus and did not show the integration site or mention any types of damages owing to the genomic integration. Lentiviruses can integrate in regions that encode gene products. This integration can cause problems by disrupting some important genes, but it can also be used as an important tool for studying gene function by observing the effects of insertional mutagenesis^11^.

## Results

The DNA construct used to generate the lentiviral particles was successfully obtained by inserting the encoding sequence for DsRed2 fluorescent protein into pLenti6.3/V5™-TOPO (Fig. S1).

Three quimiocompetent One Shot Stbl3 *Escherichia coli* colonies incorporated the plasmid in correct insert orientation (Fig. S2). The presence of the desired encoding sequence was confirmed by DNA sequencing (Fig. S3). The lentiviral particles were generated at a titre of 6.5 × 10^5^ TU/mL determined in HeLa cells; they were used to genetically modify Nile tilapia cells to achieve transgenesis.

**Figure 1:**
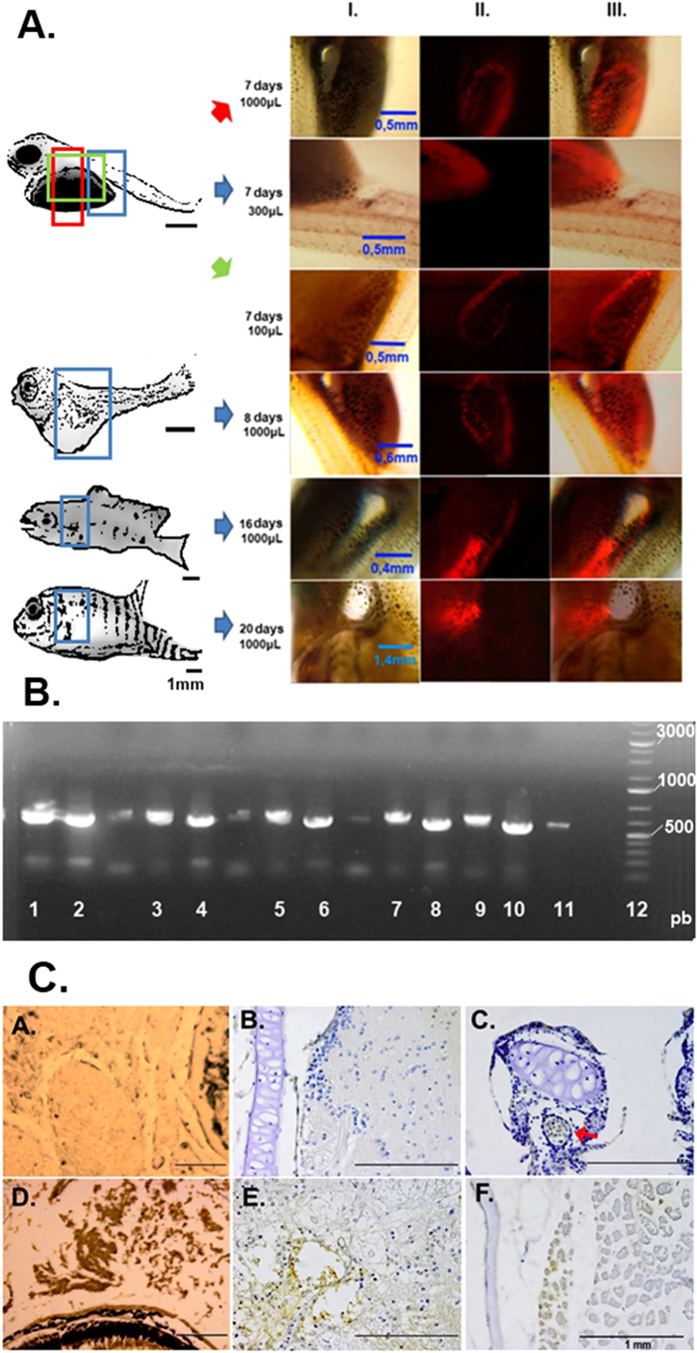
(**A**) Fluorescence microscopy images of Nile tilapia developed from transduced eggs. At the left is a schematic drawing of the fish’s full body (scale bar representing 1 mm in each drawing) with approximate areas corresponding to the fluorescence images highlighted. Next to the fluorescence images is indicated the time after exposure to the lentiviral particles and the volume of lentiviral stock used. Column I is the brightfield image, column II the fluorescence at the TRITC filter, and column III the merged images. (**B**) Agarose gel electrophoresis of PCR assay for the identification of DsRed2 mRNA in Nile tilapia developed from eggs exposed to different concentrations of lentiviral particles. 1 and 2: fish transduced with 6 × 10^5^ TU; 3 and 4: fish transduced with 3 × 10^5^ TU; 5 and 6: fish transduced with 1.8 × 10^5^ TU; 7 and 8: fish transduced with 1.5 × 10^5^ TU; 9 and 10: fish transduced with 6 × 10^4^ TU; 11: positive control, sequence amplified from the plasmid DNA pDsRed2; and 12: GeneRuler ladder 100 pb (Life Technologies). (**C**) Immunohistochemical localization of DsRed2 positive cells in Nile tilapia larvae. a. Non-stained tissue from control animal showing the eye region (without haematoxylin counterstain); b. Connective tissue of control animal stained with haematoxylin; c. Blood vessel (red arrow) near cartilage of infected animal showing DsRed2 positive cells counterstained with haematoxylin; d. Non-stained tissue from DsRed2 animal showing positive cells near the eye region; e. Conjunctive tissue of DsRed2 animal stained with haematoxylin; f. Skeletal muscle of DsRed2 animal stained with haematoxylin.

In the first strategy, the newly fertilised eggs were transduced. The fish that developed from the eggs exposed to lentiviral particles expressed the red fluorescent protein as a mosaic (Fig. 1A).

The size and intensity of fluorescent areas were related to the volume of lentiviral particles used and the time advance in a direct way: with increasing volume of viral particles and as the time advanced, the size and intensity of the fluorescent areas increased.

The mRNA for the DsRed2 fluorescent protein was present in fluorescent mosaic fish at all lentiviral particle concentrations (Fig. 1B), but the band was more intense in animals developed from eggs exposed to the higher concentration.

Through immunohistochemical assay, it was observed that the larvae developed from eggs exposed to the maximum lentiviral concentration presented a disseminated DsRed2 expression, as illustrated in the blood cells (Fig. 1Cc), eye region (Fig. 1Cd), connective tissue (Fig. 1Ce), and skeletal muscle (Fig. 1Cf - brownish areas), which is in accordance with the fact that the promoter used in DNA construction was the CMV promoter, a strong and non-tissue-specific promoter.

In order to access the main genomic integration site, nested PCR was performed. The prominent products of the nested PCR (Fig. S4) revealed that for fish that suffered premature death, the site of viral LTR integration was the phosphatidylinositol-3-kinase enzyme class 2, alpha polypeptide gene (*PI3Kc2α*) (Figs S5 and S6). In fish that survived, the insertion occurred in non-encoding regions (Fig. S7).

**Figure 2:**
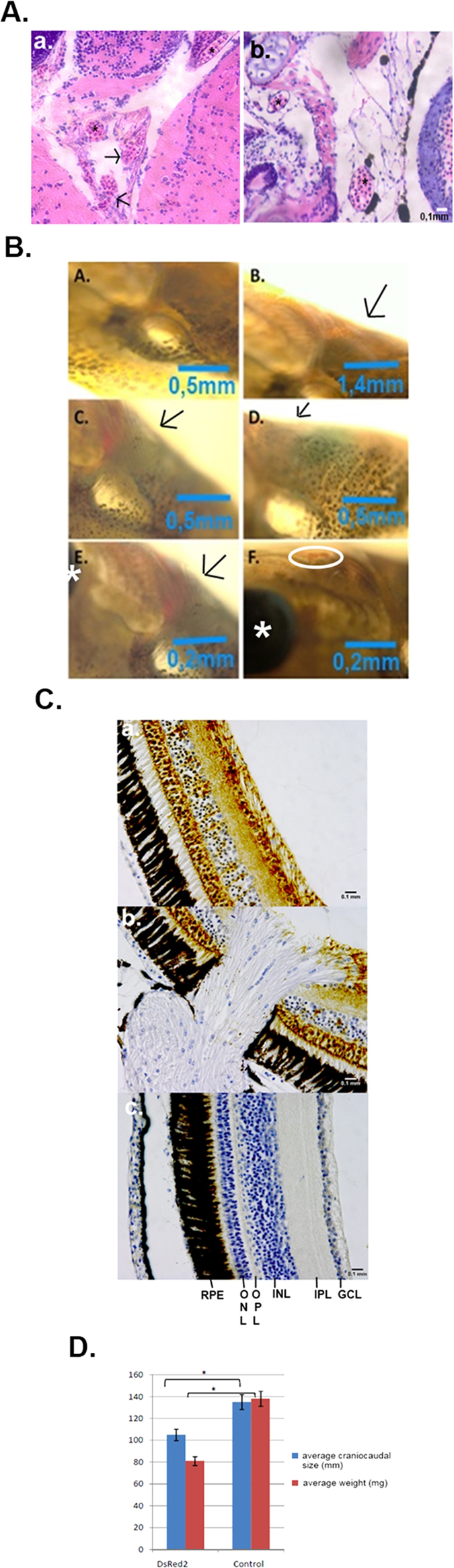
(**A**) Histology of blood vessels showing bleeding in DsRed2 fish. a. DsRed2 fish b. Control fish. The asterisk highlights some blood vessels with spaces inside that are empty in the wildtype, but are clearly congested in DsRed2 fish. (**A**) The arrows indicate blood vessels pouring out their contents. (**B**) Microscopic evaluation of DsRed2 Nile tilapia at 20 days of age. A and B: Region (highlighted by the arrow) near the eye of wildtype fish. C–F: DsRed2 fish with a purplish region and intense red blood vessels in the same location that A and B represent; in E, magnification of the hyperemic region next to the eye (asterisk); and in F, congested vessel (inside the white circle) near the bleeding area next to the eye. **(C**) Immunohistochemical detection of VEGF expression in the Nile tilapia eye region. GCL (ganglion cell layer); IPL (inner plexiform layer); INL (inner nuclear layer); OPL (outer plexiform area); ONL (outer nuclear area); RPE (retinal pigmented epithelium). a. DsRed2 fish that survived well; b. DsRed2 fish that died prematurely. c. Wildtype fish. (**D**) The size and weight of prematurely dead Nile tilapias were compromised by DsRed2 insertion into the PI3Kc2α encoding region as compared to wildtype fish (control). *indicates statistical significance by Student’s t test (p < 0.05).

The fish that suffered premature death (~67%) died from bleeding (Fig. 2A and B). Immunohistochemistry using the rabbit polyclonal antibody for VEGF receptor 1 revealed how the eye structure was compromised in DsRed2 fish that suffered premature death (Fig. 2C). These fish also showed a reduction in body weight and size (Fig. 2D).

The second genetic modification strategy was performed in SSCs exposed to lentiviral particles. Isolated Nile tilapia SSCs can be phenotypically identified by the expression of some early germ cell markers such as Vasa and Nanos2^12^ (Fig. S8A). The SSCs could be transduced by the lentiviral particles and DsRed2 fluorescence detected 24 h later. Thus, approximately 10% of the cells were already fluorescent before antibiotic (Blasticidin) selection (Fig. S8B and S8C). One month after antibiotic selection, the fluorescence level in culture increased to approximately 97% (Fig. S9), and the cells genomic DNA contained the DsRed2 encoding sequence (Fig. S10).

**Figure 3:**
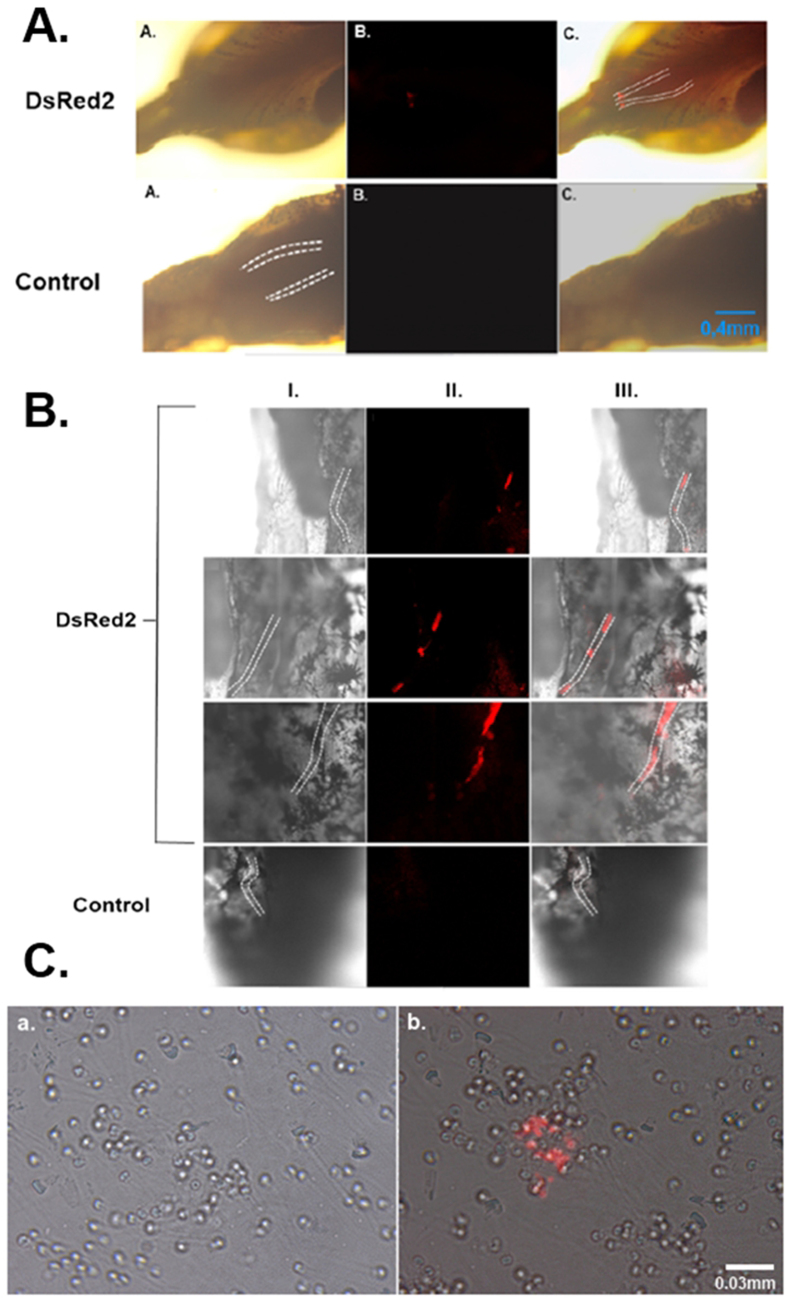
(**A**) Fluorescence microscopy of transplanted Nile tilapia larvae 7 days after transplantation. The genital ridge area is highlighted with dotted lines. Incorporation of DsRed2 positive spermatogonia were observed in genital ridge of transplanted larvae (DsRed2 B and C). No fluorescence was detected in non-transplanted controls. (**B**) Confocal microscopy of Nile tilapia gonads. Colonization of DsRed2 positive germ cells was observed in transplanted fish gonads (DsRed2 II and III). Control gonads showed no fluorescence (Control II). In column I brightfield images; column II fluorescence images at TRITC filter; column III merged images. (**C**) Fluorescence microscopy of Nile tilapia spermatozoa obtained from transplanted male. a. non-transplanted fish. b. transplanted fish.

Some previous reports have demonstrated that fish testicular germ cells, specifically SSCs, can migrate and colonize sexually undifferentiated embryonic gonad and produce functional gametes (Okutsu *et al*. 2006, Farlora *et al*. 2014, Morita *et al*. 2015). Seven days after intracoelomic injection of transduced SSCs, it was already possible to observe fluorescence related to the transduced and transplanted cells near the recipient genital ridge area (Fig. 3A).

One month after transplantation, transduced Nile tilapia SSCs colonised the gonad and the fluorescent area increased in dimension and intensity, being easily observed by confocal microscopy (Fig. 3B).

Eighty percent of the transplanted males produced genetically modified sperm after achieving sexual maturity, expressing DsRed2 fluorescent protein (Fig. 3C) at variable percentages (~5% to ~77%) (Fig. S11). Male and female fish that developed from transplanted larvae also contained the DsRed2 transcript in their gonads and/or gametes (Fig. S12).

**Figure 4:**
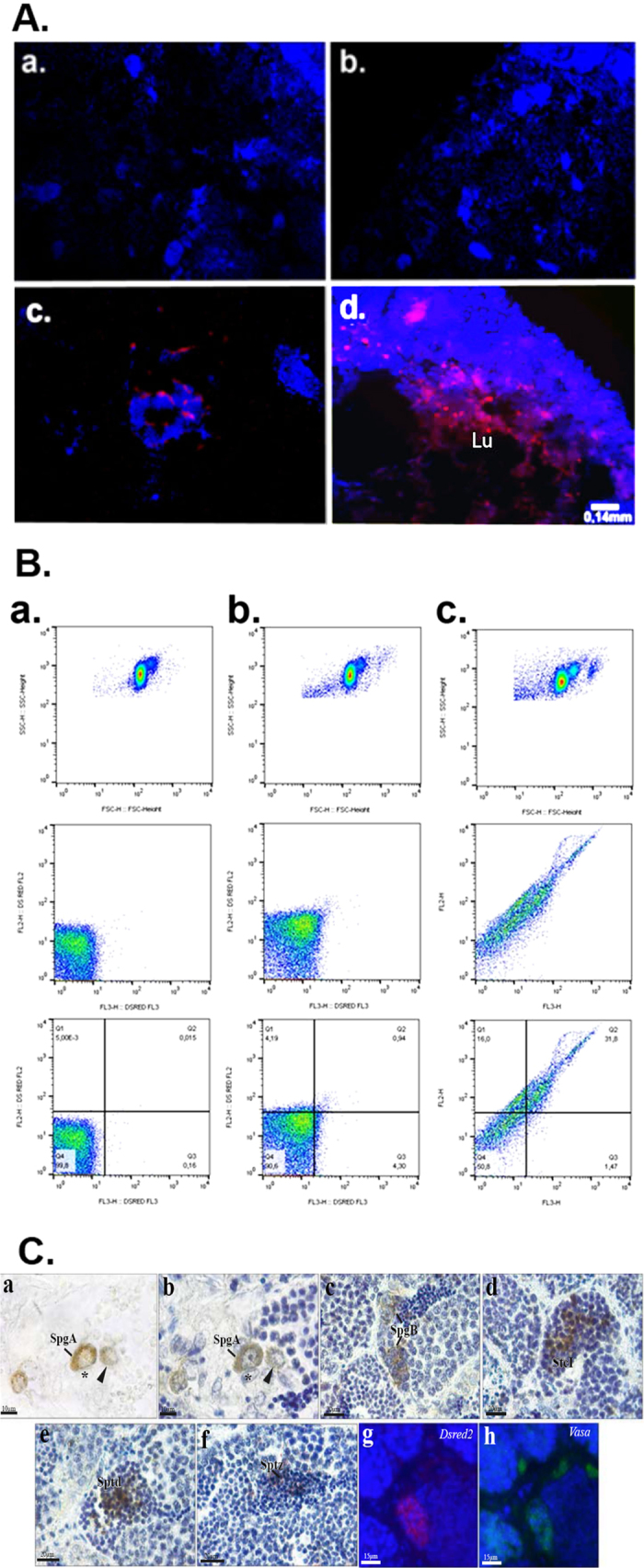
(**A**) Fluorescence microscopy of adult Nile tilapia testis injected with lentiviral particles. a-b Non-injected fish (control, respectively at 24 hours and 7 days after injection lacking lentiviral particles). c-d Injected fish (respectively 24 hours and 7 days after infection). Lu: Lumen of seminiferous tubule. (**B**) Flow cytometry panels of sperm from male fish, one week after the first evaluation. a. Wildtype fish gametes. b. *In vivo* transduced fish which had ~52% of fluorescent gamete in the first week analysis – one week later it presented only ~9%. c. Transplanted fish which had ~77% of fluorescent gamete in the first week analysis – one week later it still presented ~49%. (**C**) Immunohistochemical localization of DsRed2 positive cells in Nile tilapia testis after injection of lentiviral particles. Undifferentiated type A spermatogonia (SpgA) show clear cytoplasmic staining that can be visualized in the presence (a) or absence (b) of hematoxylin counterstain. DsRed 2 positive (arrowheads) and negative (*) Sertoli cells are also observed. c–f. Spermatogenic cysts showing DsRed2 positive germ cells in different phases of development such as type B spermatogonia (SpgB), primary spermatocytes (SpcI), spermatids (Spt) and spermatozoa in the lumen of the seminiferous tubule. g-h. Immunofluorescent staining of adjacent sections evidencing that DsRed2 positive germ cells (g, red) also express Vasa protein (h, green). Nuclei of testicular cells are stained with DAPI (g-h, blue).

The third strategy was based on genetically modified gametes exposed to lentiviral particles by i*n vivo* transduction of Nile tilapia spermatozoa. The *in vivo* transduction occurred in adult Nile tilapia testis, and 24 h after injection it was already possible to notice DsRed2 fluorescence that increased as the time advanced. DsRed2-positive cells were also observed in the lumen of the seminiferous tubules, indicating the presence of modified spermatozoa (Figs 4A and S13). The mRNA of DsRed2 was also detected in the testes and collected sperm 24 h after injection (Fig. S14).

After 7 d, the sperm from the infected animals was analysed through flow cytometry and showed the expected fluorescence. Seventy percent of the animals presented the genetic modification, and the maximum fluorescence achieved was approximately 52% (Fig. S15). Immunohistochemical analysis of Nile tilapia testis 7 days post lentiviral injection allowed the identification of transduced germ cells at different phases of spermatogenic process such as undifferentiated type A spermatogonia, type B spermatogonia, primary spermatocytes, spermatids and spermatozoa in the lumen of the seminiferous tubule. Furthermore, DsRed2 positive germ cells also express Vasa protein. Remarkably, DsRed2 positive and negative Sertoli cells were observed.

## Discussion

The fish that developed from eggs exposed to lentiviral particles were mosaic animals (Fig. 1A). This is a common feature when using newly fertilised eggs, particularly because the first cleavage occurs quickly in Nile tilapia egg, taking a maximum of 2.5 hours at 21 °C^13^, and genomic integration can take longer than this to occur. However, egg transduction by lentiviral particles can allow generation of mosaic gonads and genetically modified gametes (Fig. S16).

The integrated genomic site in animals that suffered premature death was in the phosphatidylinositol-3-kinase enzyme class 2, alpha polypeptide (PI3Kc2α) gene. This helps explaining why fish that suffered premature death (~67%) died from bleeding (Fig. 2A and B). The enzyme PI3Kc2α plays a well-known role in angiogenesis and in the establishment of vascular barrier; lack of this protein can cause vessel disruption, especially in abundantly vascularised areas such as the eyes^14^.

Impairment in the growth of DsRed2 fish could also be caused by the interruption of the PI3Kc2α gene. In mouse embryos, lack of this enzyme causes defects and eventually lethality as a result of defective vasculogenesis^14^ and defective primary cilium organization^14^. In fish, this enzyme could also play the same role, since gene interruption in the Nile tilapia larvae caused vessel disruption, congestion, bleeding, impaired growth, and premature death. The eye, a highly vascularised area, suffered major effects. This class II phosphoinositide 3-kinase is one of the downstream targets of the activated epidermal growth factor (EGF) receptor in human A431 cells^15^, and is necessary for primary cilium elongation *in vivo*^16^.

These findings pave the way for future studies of this enzyme’s overexpression or co-expression with growth hormone (GH) to generate a transgenic fish that grows faster than expected. Some transgenic animals overexpressing GH present impairment of cardiac function^17^. GH-transgenic juvenile zebrafish, for example, induce anaerobic metabolism because, unlike wild zebrafish, they are not able to sustain an aerobic metabolism when subjected to low-oxygen conditions; their pumping of blood and transporting of oxygen are impaired^18^. It is also interesting to mention that although it is already known that GH can stimulate angiogenesis, this hormone alone is insufficient to generate new blood vessels^19^.

Thus, the GH-PI3Kc2α DNA construct may offer better transgenic fish with sustained angiogenesis to achieve growth in a more stable way. Overexpression of PI3Kc2α alone may be able to create transgenic fish with faster growth and without unfavourable phenotypes commonly observed under hypoxia.

Using the *in vivo* transduction strategy, 70% of the animals presented genetic modification of the sperm, and the maximum fluorescence achieved was approximately 52%; values lower than those obtained through SSC transplantation (80% and ~77%, respectively).

The strategy of genetically modifying SSC, besides offering a higher success rate (Fig. S17) in obtaining transgenic gametes (favouring the generation, for example, of transgenic animals through the use of these gametes in *in vitro* fertilization), also results in animals with sustained production of modified gametes. Both methodologies mentioned work well in producing transgenic gametes, but *in vivo* transduction results in fish that cannot maintain a high level of fluorescence in the sperm, like the strategy involving SSC *in vitro* manipulation (Fig. 4B). Nevertheless, In addition to modified sperm, in *in vivo* transduced fish testes it was also possible to find type A spermatogonial stem cells and spermatids expressing DsRed2 (Fig. 4C).

In this study, we evaluated three different methodologies for genetically modifying Nile tilapia (Fig. S18). Although it was the simplest and fastest protocol, the transduction of newly fertilised eggs by lentiviral particles resulted in complications owing to non-site-directed integration in the fish genome at a very early developmental stage. SSC modification followed by transplantation is a strategy that generates animals with sustained production of modified sperm. An alternative to this time-consuming protocol is *in vivo* transduction; however, if this technique were chosen, *in vitro* fertilization to generate full transgenic animals would be performed with fewer (percentage-wise) transgenic sperm in comparison to the SSC strategy; and also with fluorescent sperm whose production is only temporary in fish, as one week following the first analysis the sperm fluorescence was drastically reduced (from ~52% to ~9%).

The results also made it possible to observe the effects of *PI3Kc2α* gene disruption in fish: impaired development during the embryo stage, premature death, and blood vessel compromise were present in Nile tilapia, as previously reported in the literature for mice, when the insertion of lentiviral particle genetic material occurred inside the kinase encoding sequence. This paves the way for the study of the effects of PI3Kc2α overexpression or co-expression with the GH encoding sequence to generate transgenic fish, supporting increased growth and fast development in a more favourable and stable way and avoiding impairment of cardiovascular function of animals under low oxygen tension.

## Methods

### Obtainment of lentiviral particles

The lentiviral particles were obtained using the pLenti6.3/V5-TOPO TA Cloning Kit (INVITROGEN). According to the manufacturer’s protocol, the encoding sequence for DsRed2 fluorescent protein was inserted into pLenti6.3/V5-TOPO. This sequence was amplified by PCR from pDsRed2 (CLONTECH) using the primers CCGCGGATGGCCTCTTTGCTGAAG-FWD R1 and GGGCCCTCAGTTGTGGCCCAGCTT-REV R1 (56 °C) and DreamTaq DNA Polymerase (THERMO). The construct was verified with primers V5 (ACCGAGGAGAGGGTTAGGGAT) and CMV (CGCAAATGGGCGGTAGGCGTG) from the commercial kit and used to transform the quimiocompetent One Shot Stbl3 *Escherichia coli* (INVITROGEN). The transformants were selected using ampicillin (100 μg/mL), and 19 colonies were submitted to colony PCR in order to verify their insert orientation (the primers used were V5 and FWD R1). The ones containing the DsRed2 encoding sequence in correct orientation were expanded and their plasmid DNA extracted using the PureYield Plasmid Maxiprep System (PROMEGA). The presence of the desired encoding sequence was confirmed by DNA sequencing using the primers CMV and V5, BigDye Terminator v3.1 Cycle Sequencing (INVITROGEN), and Hi-Dye formamide (INVITROGEN) in a DNA ABI 3130 automatic sequencer (APPLIED BIOSYSTEMS). The lentiviral particles were generated by cotransfection into 293FT producer cells (INVITROGEN) with components of the ViraPower Lentiviral Expression System (INVITROGEN) according to the manufacturer’s protocol. The titre of lentiviral particles was determined through transduction of HeLa cells in the presence of Polybrene (SIGMA).

In order to verify that lentiviral particles were able to induce the generation of animals with genetic modification of the gonad after being in contact with newly fertilised eggs, we also developed a DNA construct containing the sequences to generate by the same protocol lentiviral particles like those previously presented but containing the codifying sequence of eGFP fluorescent protein under the control of a germline specific promoter: the Vasa (pLenti-VseGFP).

### Transduction of newly fertilised Nile tilapia eggs

#### Egg infection and DsRed2 analysis

Thirty eggs of Nile tilapia were exposed to DsRed2 lentiviral particles in Petri dishes with tank water at five different concentrations (6.0 × 10^5^ TU–1000 μL, 3.0 × 10^5^ TU–500 μL, 1.8 × 10^5^ TU–300 μL, 1.5 × 10^5^ TU–250 μL, 6.0 × 10^4^ TU–100 μL), for 24 h under agitation at 28 °C (for eGFP particles, the exposure was performed at the optimised particle number: 1.35 × 10^6^ TU). After this time, the eggs were washed with PBS and transferred to nylon sieves under constant oxygenation in tank water at the same temperature. The expression of the red fluorescent protein was assessed by fluorescence microscopy in fish from 7 to 20 d of age. Five 20-day-old fish that developed from eggs at each of the tested concentrations had their RNA extracted using Trizol (THERMO). The presence of the DsRed2 encoding sequence was analysed by RT-PCR (first strand synthesised using the oligo(dT) primer, and the second reaction performed with primers to the DsRed2 sequence (FWD-CACTACCTGGTGGAGTTCAAG; REV-GATGGTGTAGTCCTCGTTGTG). Immunohistochemistry was performed in Nile tilapia larvae using Living Colors Anti-RCFP Polyclonal Pan Antibody (CLONTECH, primary 1:1000) and Goat anti-Rabbit IgG HRP conjugate (THERMO, secondary 1:500) according to protocol previously established^12^.

#### Analysis of the integration site and VEGF expression

The site of transgene integration into Nile tilapia genome was determined for animals that suffered premature death and for those that survived well. The techniques used to perform these analyses were gene walking through unpredictably primed PCR^20^,^21^ followed by DNA sequencing. The primers used were UP-PCR1 (TTTTTTTTTTTGTTTGTTGTGGGGGTGT) and 3IN200 (TCT GCT TTT TGC TTG TAC TGG GTC TCT C) for the first PCR and UP-PCR2 (TTTTTGTTTGTTGTGGG) and 3IN100 (TTG CCT TGA GTG CTT CAA GTA GTG TGT) for the second PCR. Since the fish with premature mortality died because of bleeding, immunohistochemistry using rabbit polyclonal antibody for VEGF receptor 1 (ABCAM 1:100) as the primary antibody was also performed in these animals^12^.

### *In vitro* genetic modification of SSCs and transplantation into Nile tilapia larvae

Nile tilapia SSCs were obtained through primary culture from adult fish testis^12^,^22^,^23^,^24^, and 10^6^ cells per dish were exposed to 1 mL of lentiviral solution (titre = 6.5 × 10^5^ TU/mL). The cells were incubated at 33 °C and 5% CO_2_ for 24 h, and the aliquot was submitted to flow cytometry in order to assess the percentage of fluorescent cells before selection. Blasticidin (6 μg/mL) was added to the supplemented DMEM/F12 culture medium (High Glucose DMEM/F12 (THERMO), 10% foetal bovine serum, 0.1 mM non-essential amino acids, 6 mM L-glutamine, 1 mM sodium pyruvate, and 1% penicillin-streptomycin) and the cells were maintained for one month^12^. After this time, their fluorescence was assessed by flow cytometry. Cell genomic DNA was extracted and submitted to PCR in order to confirm the presence of the DsRed2 encoding sequence on a 1% agarose gel with Syber Safe (LIFE TECHNOLOGIES). Twenty larvae (2–3 days after hatching) were anesthetised and microinjected in the coelomic cavity with approximately 2 μL of cell suspension containing 5 × 10^4^ SSCs/μL, using a micromanipulator (NARISHIGE) and glass needles prepared with GD-1 capillaries (NARISHIGE) as previously described^25^. The red fluorescence was monitored through fluorescence and confocal microscopy, and the sperm samples from male recipients were analysed by flow cytometry and fluorescence microscopy.

### *In vivo* transduction of spermatozoa from adult fish

In adult Nile tilapia, the only non-surgical access to the testis is via the common spermatic duct that opens in the urogenital papilla through the urogenital pore. Ten 6-month-old male fish were anesthetised and received 300 μL of DsRed2 lentiviral particle solution (6.5 × 10^5^ TU) through the common spermatic duct using a glass micropipette (outside diameter 70 μm) under a stereomicroscope (OLYMPUS SZX-ILLB2-100)^26^. They were maintained for 1 week in tank water at 30 °C to stimulate spermatogenesis^27^. The gonads were removed in duplicate 24 h, 3.5 d and 7 d after injection, and immersed in Tissue-Tek O.C.T. Embedding Compound (SAKURA FINETEK). Cryomicrotome sections were washed in PBS, stained using DAPI (SIGMA), and then analysed by fluorescence microscopy in order to verify the red fluorescent protein expression in the fish testis. Twenty-four hours and 7 d after lentiviral injection, sperm were also collected by abdominal massage and submitted to flow cytometry. mRNA obtained from the fish testis and isolated sperm was analysed by RT-PCR as described above. Immunohistochemistry was also performed in transduced Nile tilapia testis using Living Colors Anti-RCFP Polyclonal Pan Antibody (CLONTECH) and anti-DDX4/Vasa (ABCAM, 1:400). Secondary Alexa Fluor488 donkey anti-Rabbit IgG (INVITROGEN, 1:500) was used for fluorescent detection.

### Ethics approval

The experiments were performing in accordance with the guidelines approved by the local ethics committee on animal use - CEUA, UFMG (protocol# 89/2012).

## Additional Information

**How to cite this article**: Tonelli, F. M. P. *et al*. Gene delivery to nile tilapia cells for transgenesis and the role of PI3K-c2α in angiogenesis. *Sci. Rep.* **7**, 44317; doi: 10.1038/srep44317 (2017).

**Publisher's note:** Springer Nature remains neutral with regard to jurisdictional claims in published maps and institutional affiliations.

## Supplementary Material

Supplementary Materials
