# Supplementary material for: Gene delivery to Nile tilapia cells for transgenesis and the role of PI3K-c2α in angiogenesis
Source: Sci Rep. 2017 Mar 20;7:44317. doi: 10.1038/srep44317 (PMC5357942; doi:10.1038/srep44317)
Supplement: Supplementary Materials [file srep44317-s1.doc]

**GENE DELIVERY TO NILE TILAPIA CELLS FOR TRANSGENESIS AND THE ROLE OF PI3K-C2α IN ANGIOGENESIS**

**Fernanda Maria Policarpo Tonelli^1,2#^, Samyra Maria dos Santos Nassif Lacerda^3#^, Marcela Santos Procópio^3^, Breno Luiz Sales Lemos^3^, Luiz Renato de França^3, 4*^, Rodrigo Ribeiro Resende^1,2*^**

^1^Cell Signaling &Nanobiotechnology Laboratory, Department of Biochemistry & Immunology, Federal University of Minas Gerais, Belo Horizonte, Brazil. [tonellinanda@gmail.com](mailto:tonellinanda@gmail.com)

^2^Nanocell Institute, Divinópolis, MG, Brazil.

^3^ Laboratory of Cellular Biology, Department of Morphology, Federal University of Minas Gerais, Belo Horizonte, Brazil. [samyranassif@hotmail.com](mailto:samyranassif@hotmail.com); [marcela.procopio@hotmail.com](mailto:marcela.procopio@hotmail.com); [brenoluizsales@gmail.com](mailto:brenoluizsales@gmail.com);

^4^National Institute for Amazonian Research (INPA), Manaus, AM, Brazil.

#These authors contributed equally to this work

*Corresponding authors: Rodrigo R Resende - Cell Signaling and Nanobiotechnology Laboratory, Department of Biochemistry and Immunology, Federal University of Minas Gerais, Av. Antônio Carlos, 6627 – Pampulha – PO box 486 – 31270-901 – N4 – room 112. Belo Horizonte, Brazil. 55(31)34092627. [rrresende@institutonanocell.org.br](mailto:rrresende@institutonanocell.org.br)

Dr. Luiz Renato de França – [lrfranca@inpa.gov.br](mailto:lrfranca@inpa.gov.br) National Institute for Amazonian

Research (INPA), Manaus, AM, Brazil.


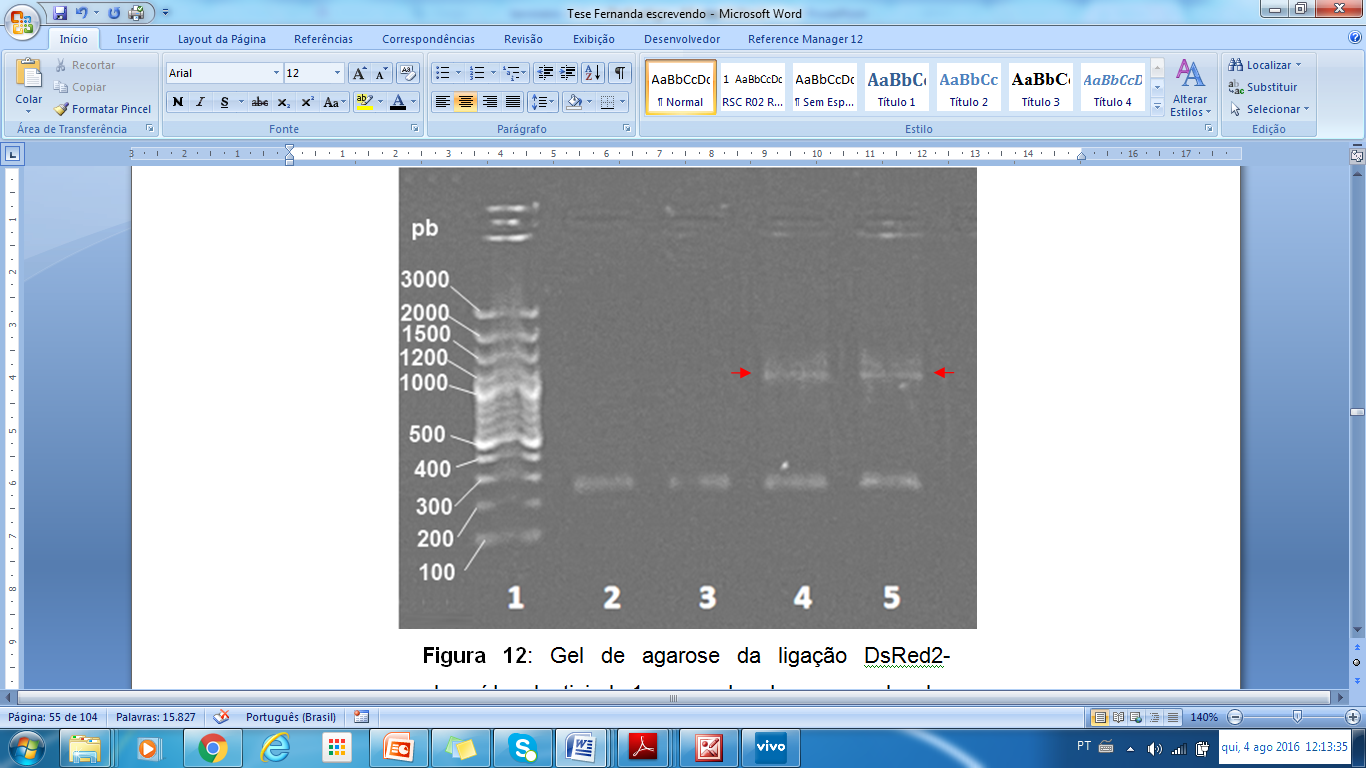


**Figure S1:** Agarose gel electrophoresis of PCR assay for the identification of DsRed2 sequence into pLenti6.3/V5™-TOPO using CMV and V5 primers. 1- the Gene Ruler 1kb ladder (Fermentas); 2-empty circularized pLenti6.3/V5™-TOPO® plasmid annealing at 56ºC; 3 - empty circularized pLenti6.3/V5™-TOPO® plasmid annealing at 62ºC; 4- ligation product annealing at 56ºC; 5- ligation product annealing at 62ºC. The arrow indicates the expected band size (~1000 pb).


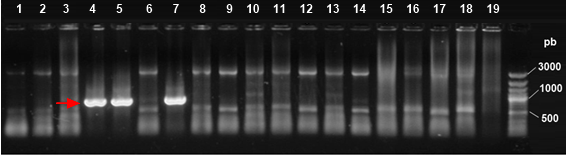
 **Figure S2**: Agarose gel electrophoresis of colony PCR products from 19 colonies using primers V5 and FWD R1. The Gene Ruler 1kb ladder (Fermentas) is presented in the right side of the figure. The arrow indicates the band correspondent to the incorporation of the insert in correct orientation (~900pb).

**
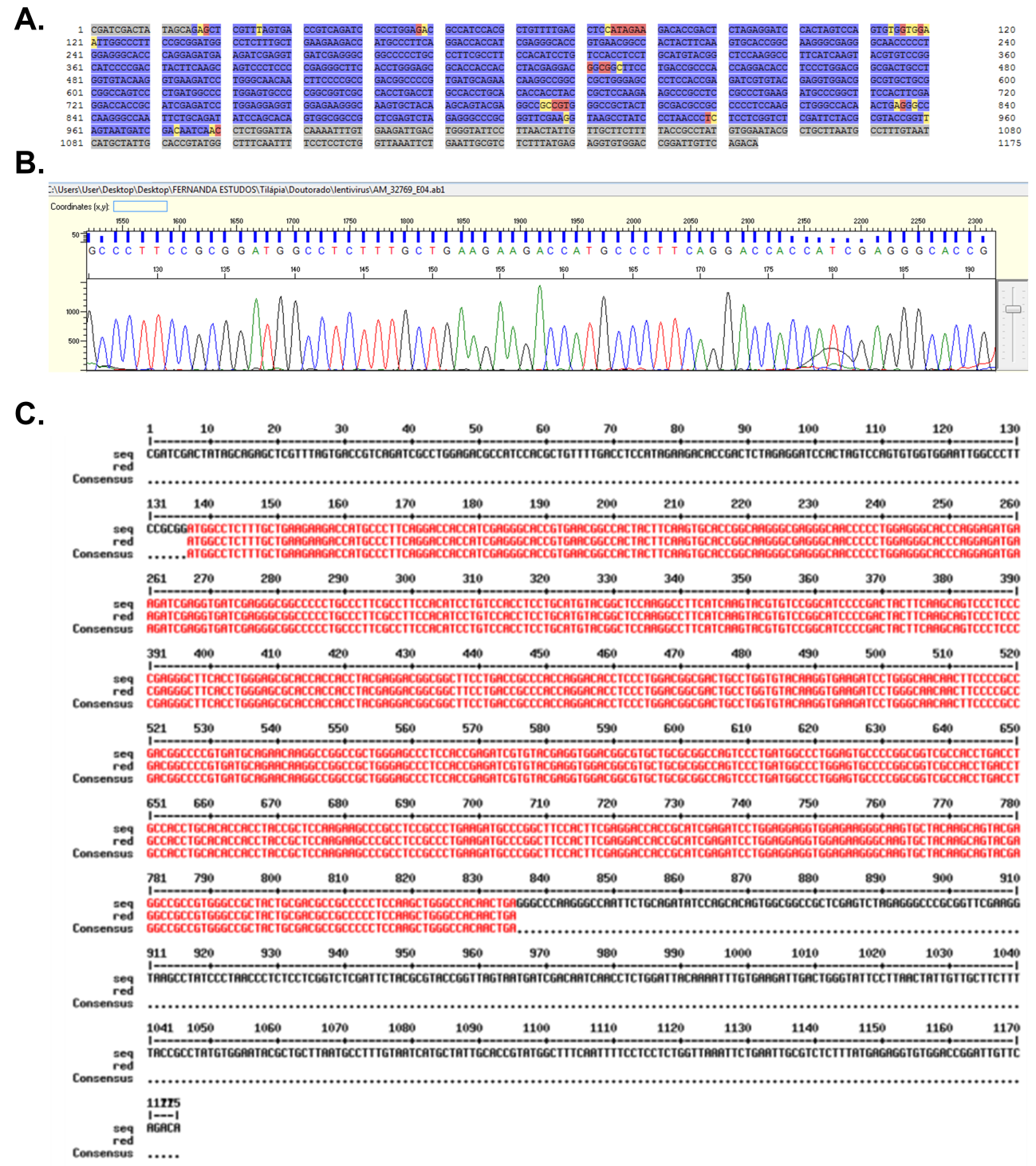
**

**Figure S3**: DNA sequencing resulted for colony 4 using the CMV primer. **A** the sequence was colored to reflect the pure base quality value: blue-high, yellow-medium, red-low. **B** sequence chromatogram from the base pair 125 to 191. **C** sequencing alignment resulted with the DsRed2 sequence.


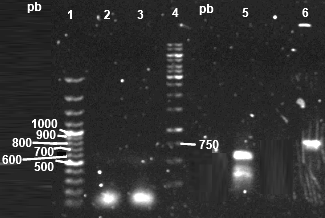


**Figure S4:** Agarose gel from the nested PCR (gene walking). 1-GeneRuler Ladder 100 pb (Fermentas); 2 and 3 – non-integrated fish; 4- GeneRuler Ladder 1kb (Fermentas); 5- animal that survived well; 6- fish that suffered premature death.


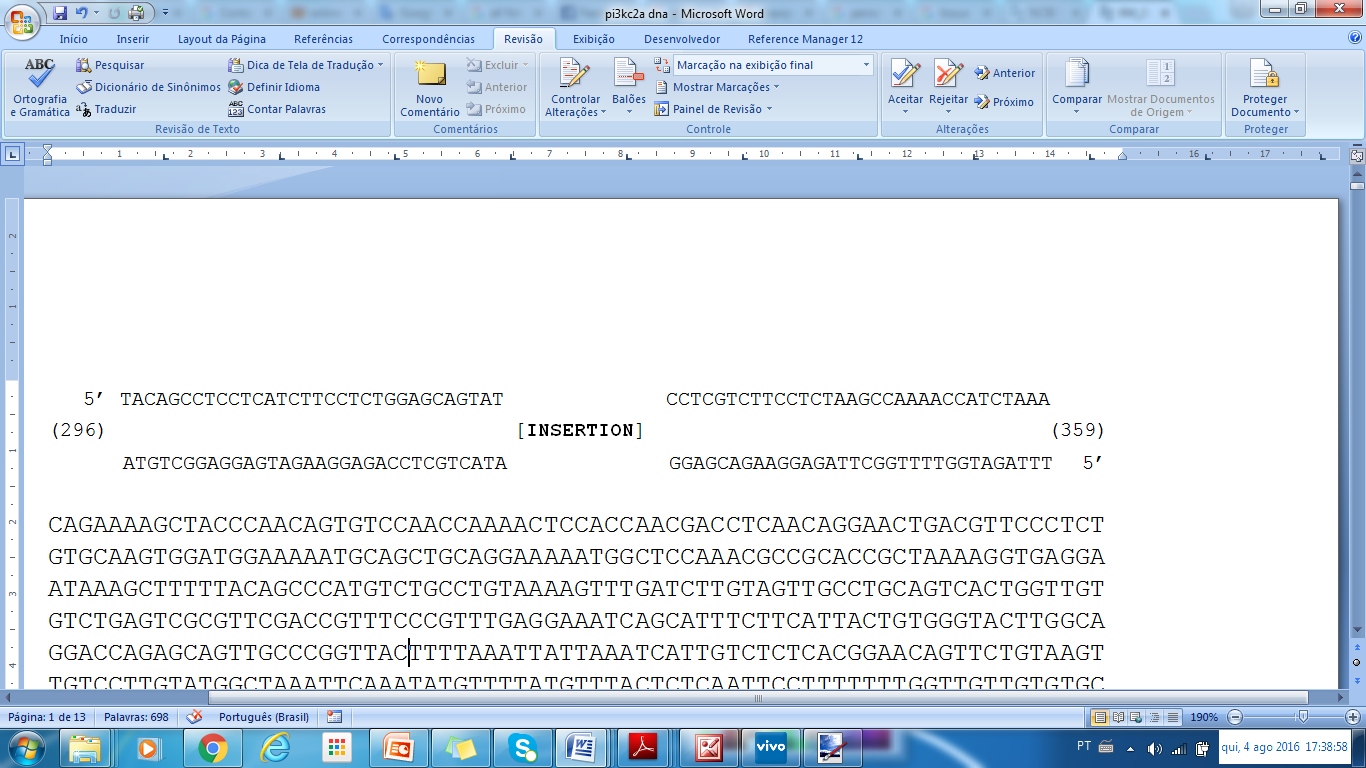


**Figure S5:** Schematic representation of the insertion site of the transgene inside PI3Kc2α Nile tilapia gene that was comprised between the base pairs 327 and 328.


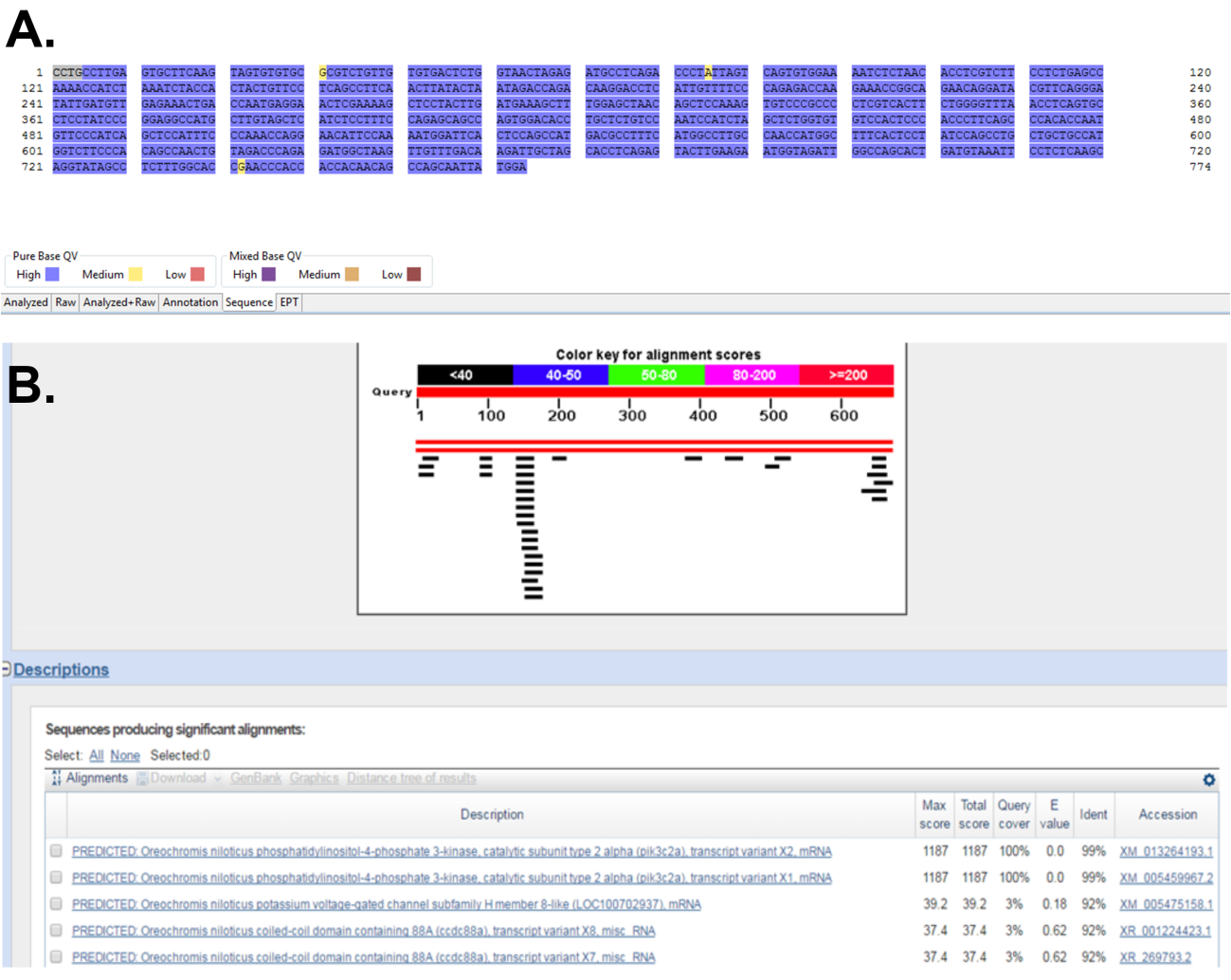


**Figure S6:** Genomic integration site in fish that died prematurely. **A**DNA sequencing result. **B**BLAST of the result indicating insertion inside the gene of PI3Kc2α.


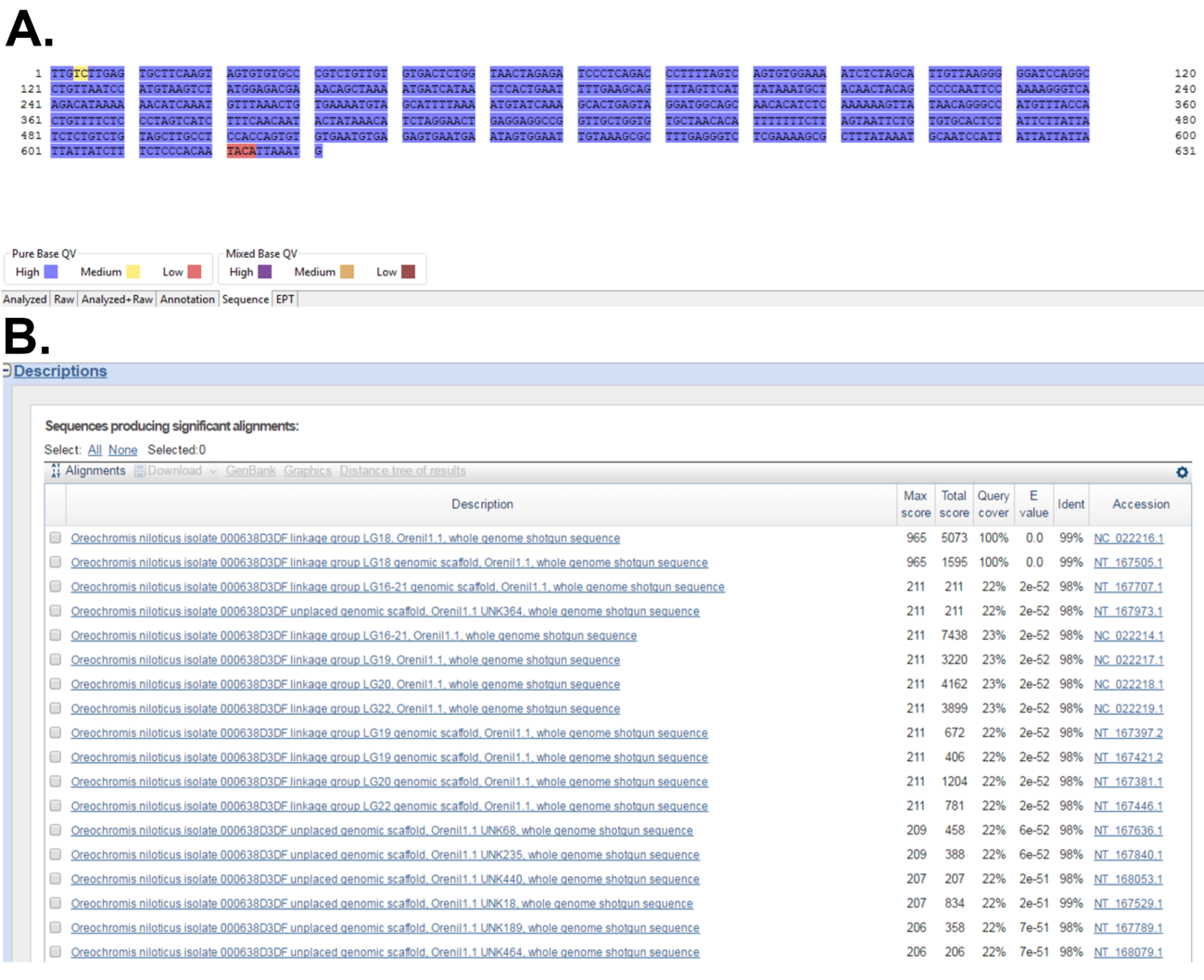


**Figure S7:** Genomic integration site in fish that survived healthy. **A** DNA sequencing result for the prominent fragment of 631 pb from Figure S2. **B** BLAST of this result indicating that the insertion occurred in a non-encoding region.


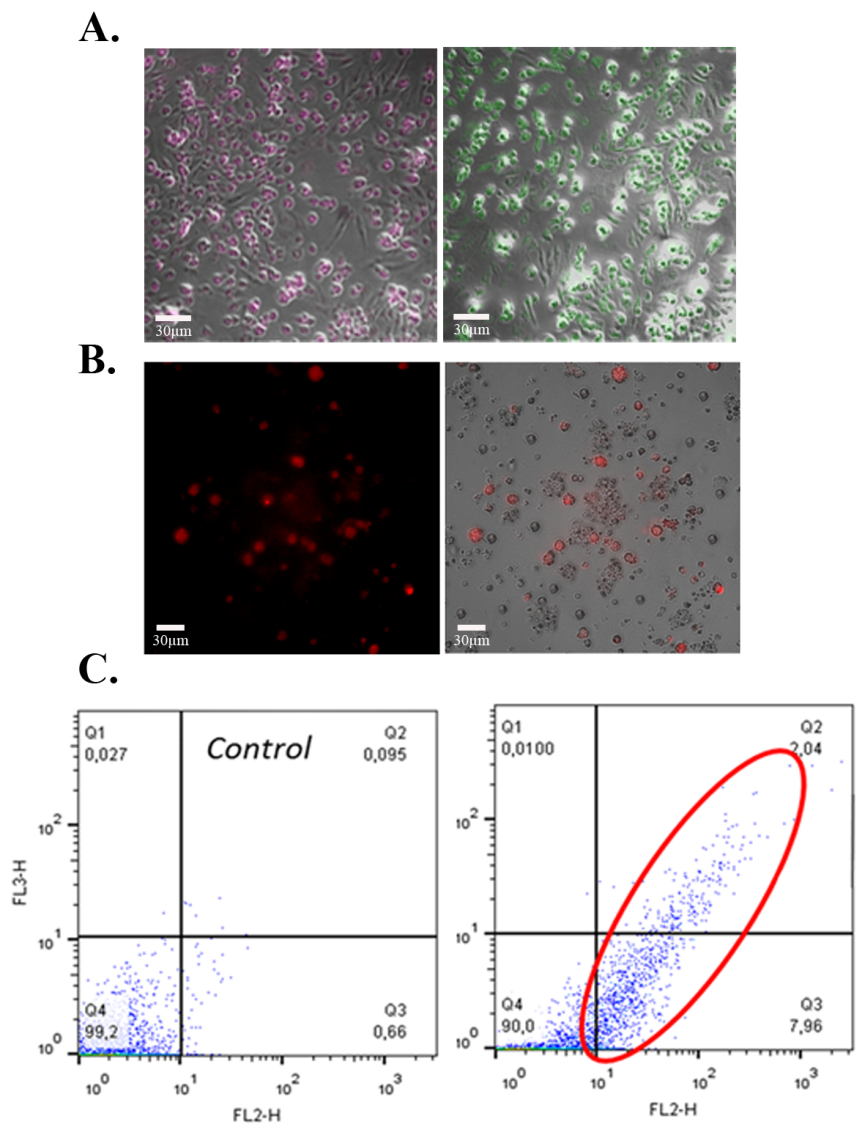


**Figure S8:** **A.** Immunohistochemical evaluation of Nile tilapia SSCs. The expression of Vasa (pink) and Nanos2 (green) can phenotypically identify these cells. **B.** Transduced Nile tilapia SSCs before antibiotic selection. At left, fluorescent microscopy showing DsRed2 positive cells. At right, merged brightfield and fluorescent image **C.** Flow cytometry panels for transduced SSCs fluorescence level without antibiotic selection.

**
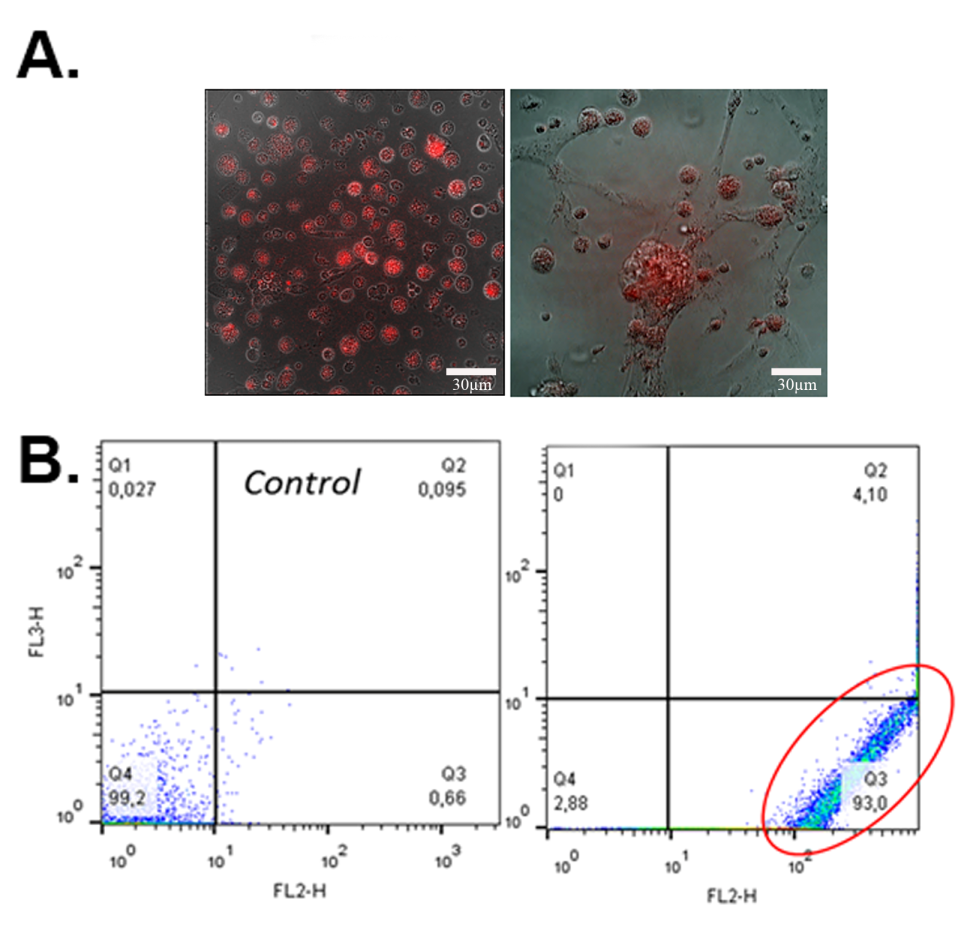
**

**Figure S9: A.** Transduced Nile tilapia SSCs one month after antibiotic selection. Merged brightfield and fluorescent images show high number of DsRed2 positive cells isolated (red, in the left) or forming typical colonies (red, in the rigth) . **B.** Flow cytometry panels for transduced SSCs fluorescence level one month after of culture under antibiotic (Blasticidin) selection.


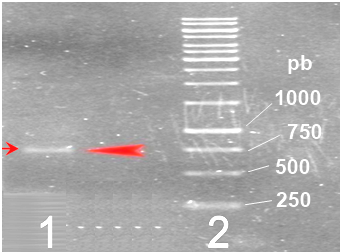


**Figure S10**: Agarose gel of PCR product (primers R5 FWD -CTGCAGATGGCCTCTTTGCTGAAG and R5 REV – GAGCTCATGGCTGATTATGATCTA) from transduced SSCs one month after culture and Blasticidin selection. The arrows indicate in lane 1 the fragment of ~740pb related to DsRed2 encoding sequence.In lane 2, GeneRuler ladder 1kb (Fermentas).


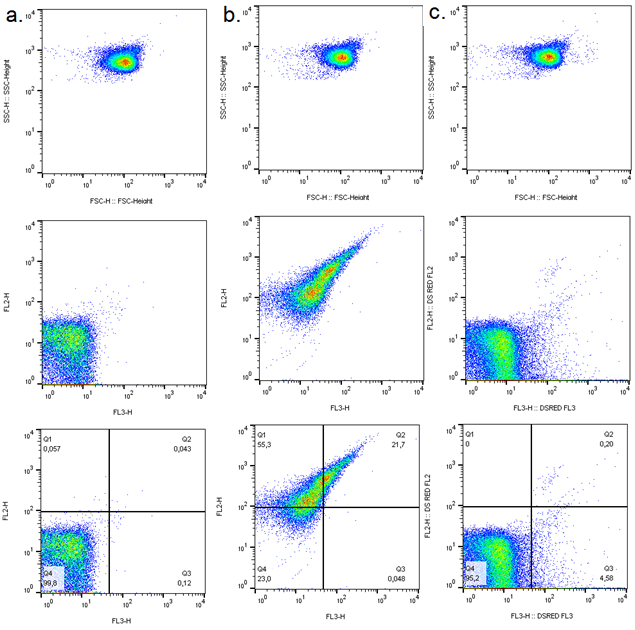


**Figure S11:** Flow cytometry of sperm from male recipient fish developed from the larvae transplanted with genetically modified SSCs from Nile tilapia. **A** non-transplanted fish, **B-C** transplanted fish.


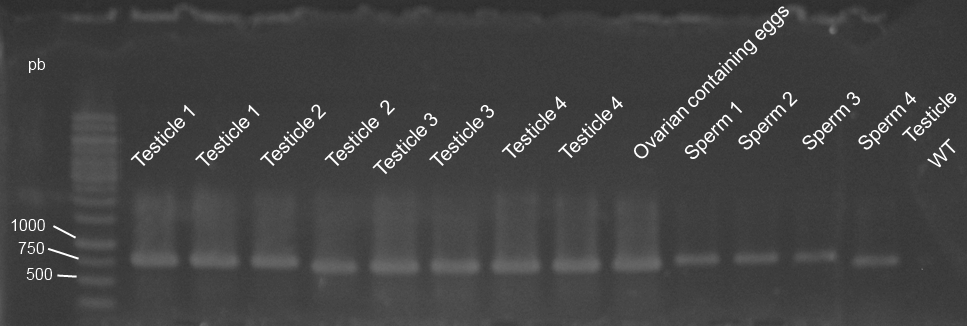


**Figure S12:** Agarose gel electrophoresis of PCR assay for the identification of DsRed2 cDNA in the gonad and/or gamete of transplanted and wildtype (WT) fish. The DsRed2 transcript was present in testis, sperm and ovary of transplanted animals investigated. In the left, GeneRuler ladder 1000pb (Life Technologies).


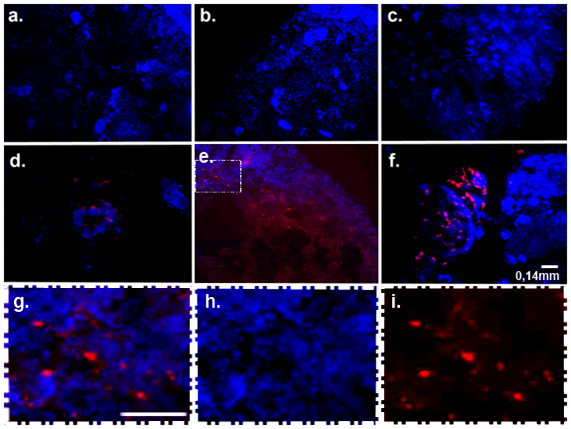


**Figure S13:** Fluorescence microscopy of adult Nile tilapia testis injected with lentiviral particles. **A-C** non-injected fish (control, respectively at 24 h., 3.5 and 7 days after injection). **D-I** injected fishes (**D**: 24hs; **E**: 3.5 days; **F**: 7 days after infection; **G-I**: higher magnification of the area highlighted in E, merged, DAPI, DsRed fields, respectively).

**
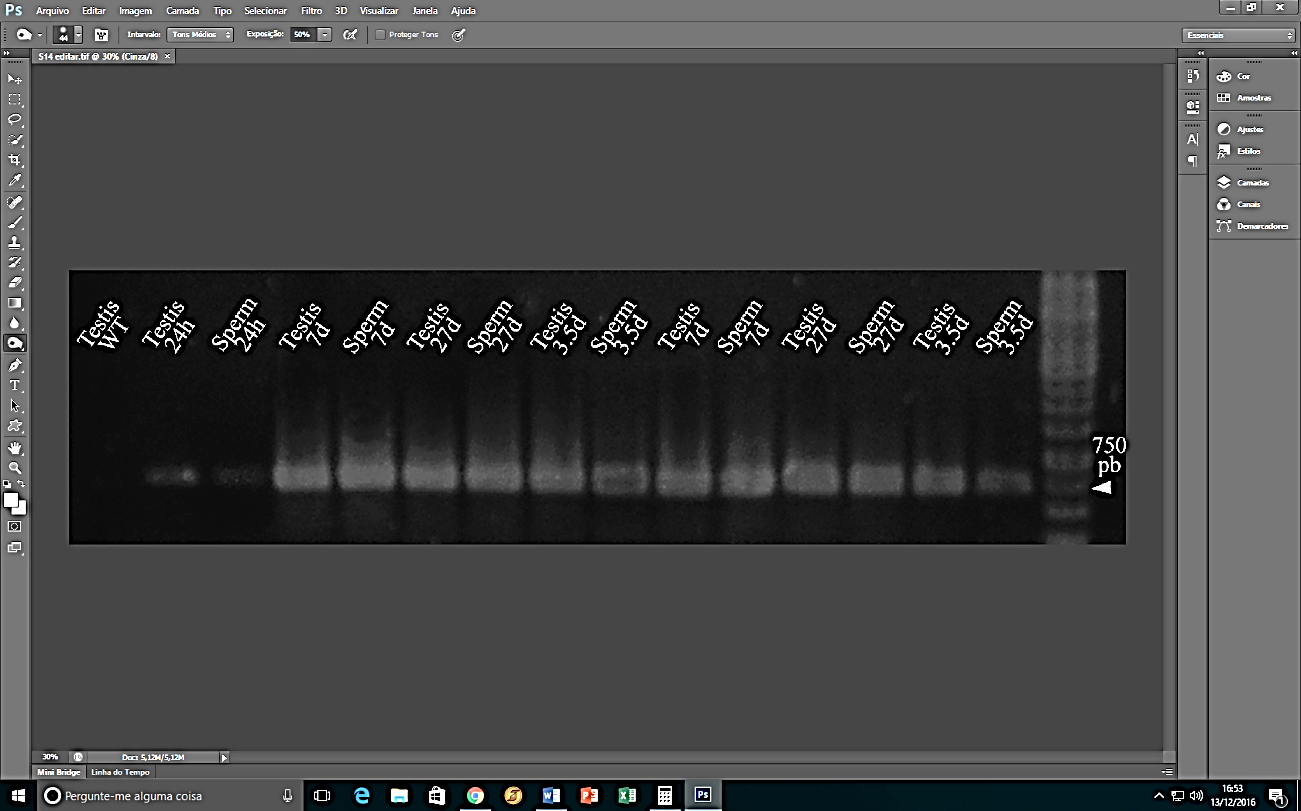
**

**Figure S14:** Agarose gel electrophoresis of PCR assay for the identification of DsRed2 cDNA in the testis and sperm of *in vivo* transduced and wildtype (WT) Nile tilapia. The DsRed2 transcript (~740 pb) was detected in all investigated samples of injected fish. In the right, GeneRuler ladder 1000pb (Life Technologies).

**
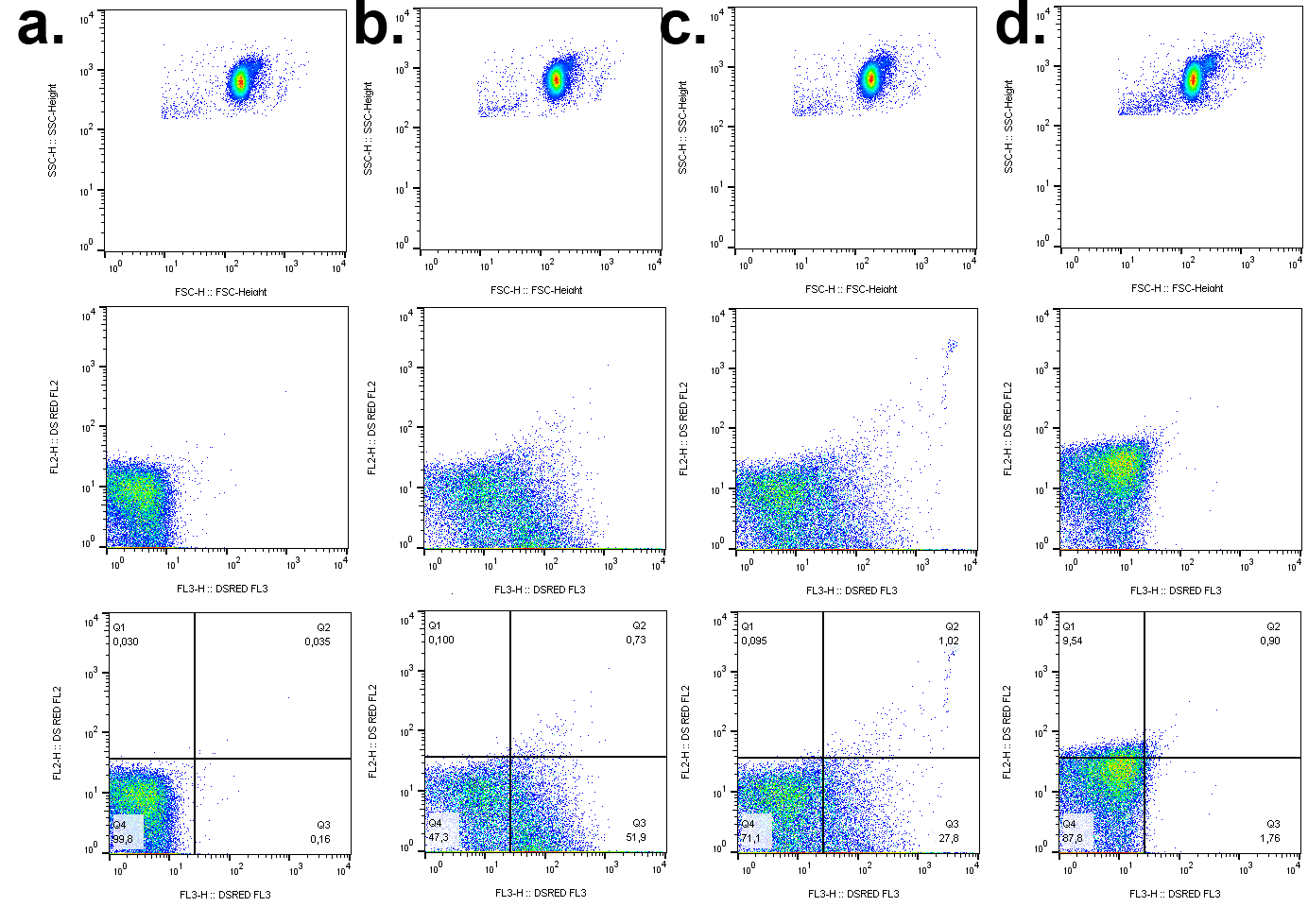
**

**Figure S15:** Flow cytometry panels of sperm obtained from male 7 days after *in vivo* transduction. **A.** non-injected fish. **B-D.** injected fish.


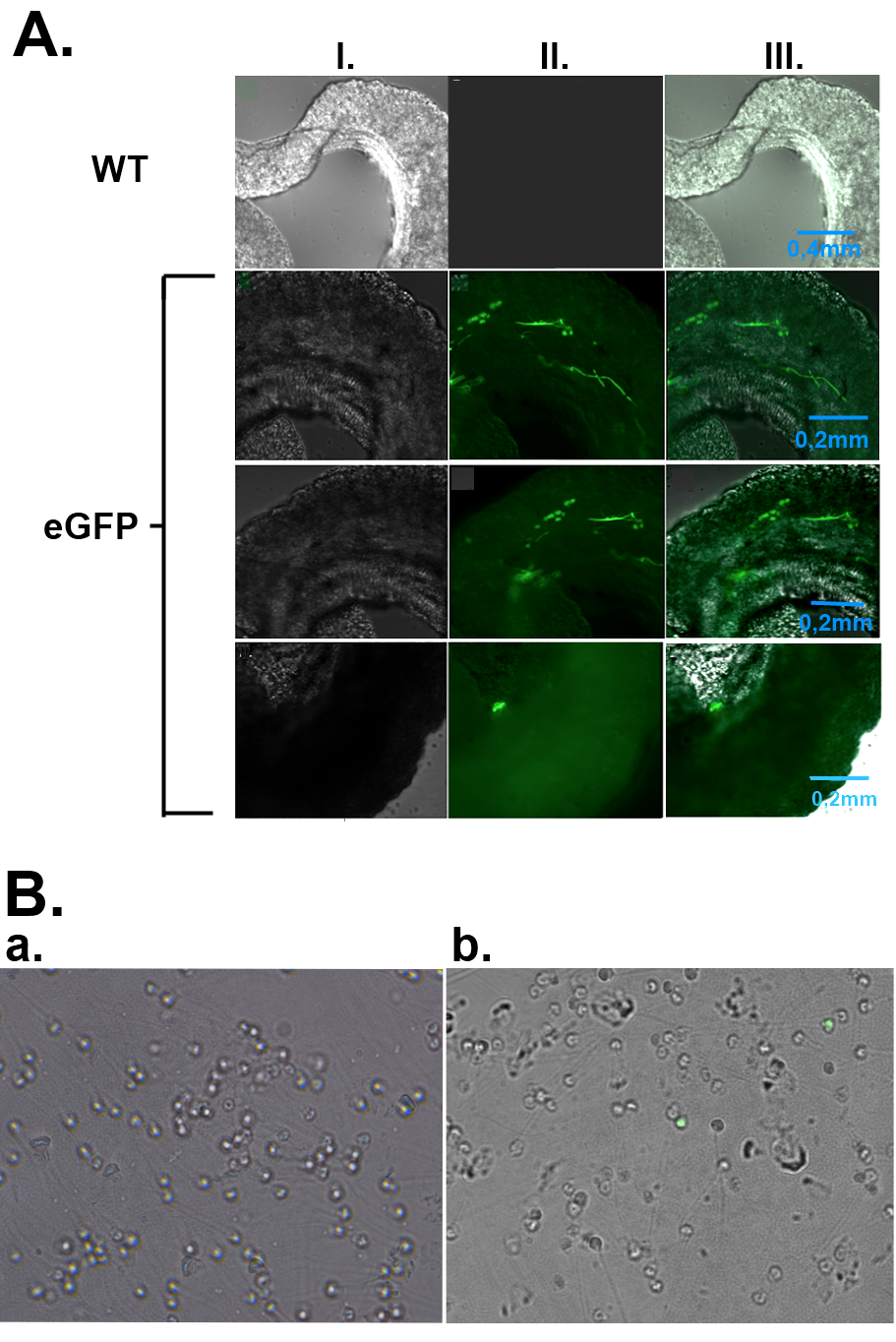


**Figure S16:** Gonad and sperm of animals developed from newly fertilized transduced with the pLenti-VseGFP construct or from WT eggs. A. Fluorescence microscopy from fragments of testicles from adult animals; I brightfield images; images from FITC filter; III merged images from I and II. B. Fluorescence microscopy of adult Nile tilapia sperm; a. from WT animals; b. from animals developed from transduced sperms.

**
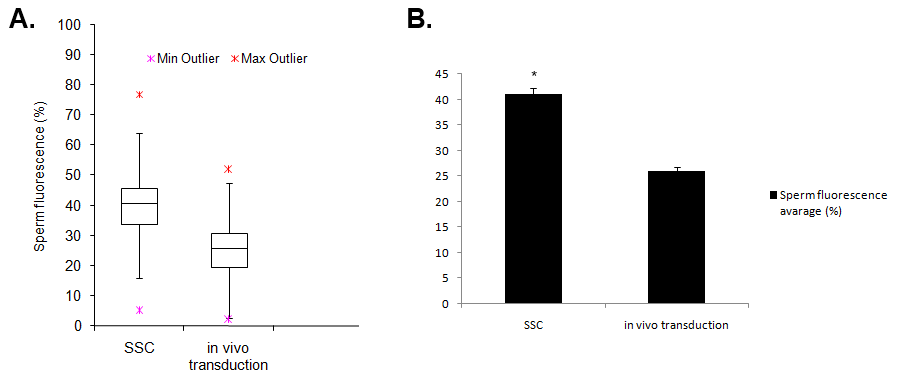
**

**Figure S17:** **A.** Boxplot analysis of the data related to the strategies involving SSCs and the in vivo transduction. **B.** Comparison of the average of sperm fluorescence percentage obtained by the mentioned methodologies (*t* test; p<0.05).

**Figure S18:** Three different methodologies to genetically modify Nile tilapia.
